# Unbalance in Iron Metabolism in Childhood Leukemia Converges with Treatment Intensity: Biochemical and Clinical Analysis

**DOI:** 10.3390/cancers13123029

**Published:** 2021-06-17

**Authors:** Monika Łęcka, Artur Słomka, Katarzyna Albrecht, Ewa Żekanowska, Michał Romiszewski, Jan Styczyński

**Affiliations:** 1 Department of Pediatric Hematology and Oncology, Jurasz University Hospital, Collegium Medicum Nicolaus Copernicus University Torun, 85-094 Bydgoszcz, Poland; lecka.monika@doktorant.umk.pl; 2 Department of Pathophysiology, Collegium Medicum Nicolaus Copernicus University Torun, 85-094 Bydgoszcz, Poland; artur.slomka@cm.umk.pl (A.S.); zorba@cm.umk.pl (E.Ż.); 3 Department of Oncology, Hematology, Bone Marrow Transplantation and Pediatrics, Medical University of Warsaw, 02-091 Warsaw, Poland; katarzyna.albrecht@wum.edu.pl (K.A.); michal.romiszewski@uckwum.pl (M.R.)

**Keywords:** children, acute leukemia, hematopoietic cell transplantation, iron metabolism

## Abstract

**Simple Summary:**

In children undergoing therapy for acute leukemia or after hematopoietic cell transplantation, the following iron metabolism parameters were analyzed in the context of iron overload: (1) parameters measuring functional and storage iron pools: non-transferrin-bound iron (NTBI) and labile plasma iron (LPI) levels, iron, transferrin, total iron-binding capacity, ferritin, ferritin heavy and light chains; (2) proteins regulating iron absorption and its release from tissue stores: hepcidin, soluble hemojuvelin, soluble ferroportin-1; (3) proteins regulating the erythropoietic activity of bone marrow: erythroferrone, erythropoietin, soluble transferrin receptor. It has been shown that the occurrence of NTBI and LPI in the circulation and the intensification of disturbances in iron metabolism were associated with the intensity of anti-leukemic treatment and were the highest in the transplant group followed by the acute leukemia after treatment and de novo groups. In patients after transplantation, the most significant changes were found in NTBI, LPI, iron, ferritin, hepcidin, and ferroportin-1 levels.

**Abstract:**

Objective: The aim of this study was to evaluate non-transferrin-bound iron (NTBI) and labile plasma iron (LPI) levels and other parameters of iron metabolism in children undergoing therapy for acute leukemia or after hematopoietic cell transplantation (HCT), in the context of iron overload. Patients: A total number of 85 children were prospectively included into four groups: controls, acute leukemia de novo, acute leukemia after intensive treatment, and after HCT. Methods: The following iron metabolism parameters were analyzed: (1) parameters measuring functional and storage iron pools: NTBI, LPI, iron, transferrin, total iron-binding capacity, ferritin, ferritin heavy and light chains; (2) proteins regulating iron absorption and its release from tissue stores: hepcidin, soluble hemojuvelin, soluble ferroportin-1; (3) proteins regulating the erythropoietic activity of bone marrow: erythroferrone, erythropoietin, soluble transferrin receptor. Results: Intensive treatment of leukemia in children was associated with the presence of serum NTBI and LPI, which was the highest in the HCT group followed by the acute leukemia after treatment and de novo groups. In patients after HCT, the most significant changes were found in NTBI, LPI, iron, ferritin, hepcidin, and ferroportin-1 levels. Conclusions: The occurrence of NTBI and LPI in the circulation and the intensification of disturbances in iron metabolism were associated with the intensity of the anti-leukemic treatment.

## 1. Introduction

Iron overload is a common secondary complication in patients treated for acute leukemia or undergoing hematopoietic cell transplantation (HCT), resulting from frequent red blood cell transfusions [1,2,3]. Each milliliter of transfused red cells contains 0.8 mg iron [4], and thus multiple transfusions contribute to rapid iron accumulation. Transfusional iron overload increases the risk of infectious complications and the proliferation of malignant cells [5], associated with an increased risk of veno-occlusive disease (also known as hepatic sinusoidal obstruction syndrome), incidence of graft-versus-host disease, non-relapse mortality, and reduced overall survival [6]. The evidence of the deleterious effect of iron burden on the outcomes of transplantation comes mainly from studies analyzing serum ferritin as a marker for iron overload.

In the case of iron overload, the iron-binding ability of transferrin is highly exceeded, and thus non-transferrin-bound iron (NTBI) appears in the blood [1,7]. NTBI usually rises markedly with the increase in transferrin saturation up to 70%, whereas a highly reactive Fe^2 +^ species of NTBI referred to as labile plasma iron (LPI) increases simultaneously [1,7]. Therefore, the risk of an imbalance in the entire iron homeostasis should be taken into account. The toxicity of iron results from the Fe^2 +^ forms of iron, which are highly reactive and cause rapid oxidant damage of proteins and DNA, permanently changing the structure of proteins and genetic material [7,8]. This process is mainly caused by the excess of NTBI and its fraction LPI [2]. 

NTBI is a toxic, low-molecular weight fraction of iron, which is usually detectable in iron-overloaded patients. NTBI can also be present in patients during cytotoxic chemotherapy, contributing to organ damage following chemotherapy [9]. NTBI possibly reflects a significant disturbance in iron utilization which is related to its release from dying cells in bone marrow and possibly other tissues, as well as liver toxicity and decreased transferrin production [10,11]. NTBI has a heterogenous character related to the degree, duration, and etiology of iron overload. LPI is the most toxic fraction of NTBI components and represents deleterious, organ-penetrating redox-active forms of iron. It is a labile and chelatable form that can induce tissue iron overload and permeate into organs, causing their damage [12,13]. LPI itself, more than NTBI, can provide a direct insight into chelatable iron which is not explained by other diagnostic tests of iron metabolism such as ferritin and transferrin saturation [12,14]. 

Iron metabolism is also regulated by a number of proteins. The principal regulator of iron homeostasis is hepcidin, a 25-amino acid protein secreted primarily by hepatocytes [15]. Hepcidin regulates iron absorption and release from tissue stores by targeting ferroportin and downregulates the entry of iron into the plasma by downregulating ferroportin, the sole cellular iron exporter [16]. Hepcidin itself is also subject to complex molecular control involving, among other factors, hemojuvelin (HJV), which, depending on its form, has opposite functions. Membrane HJV stimulates hepcidin synthesis. Still, the blood-circulating form (sHJV) significantly reduces its production [17,18]. The role of circulating ferroportin (FNP-1) remains a subject that is undergoing intense research, both in its release mechanisms from the cell membrane and its biological functions. It is known that the membrane form of this protein is a transporter involved in the transfer of iron from the cells to the bloodstream, which is degraded by the action of hepcidin. The concentration of FNP-1 in the blood seems to reflect the amount of the membrane form of this protein, just as the soluble transferrin receptor (sTfR) demonstrates the expression of the membrane receptor for transferrin.

Erythroferrone (ERFE) is the main regulator of hepcidin synthesis. Increased ERFE suppresses hepcidin synthesis, leading to mobilization of cellular iron stores and its use in heme and hemoglobin synthesis. Overproduction of ERFE and suppressing hepcidin can cause iron overload, even in non-transfused patients. ERFE can be regarded as a biomarker of ineffective erythropoiesis and a possible therapeutic target [19].

Due to frequent red blood cell transfusions, patients with acute leukemia or undergoing HCT are at increased risk of iron overload and its consequences. However, the precise mechanisms behind these processes are far from being understood. They are even less known in pediatric leukemia patients and children after HCT. Thus, the purpose of this prospective study was to evaluate NTBI and LPI levels in children undergoing therapy for acute leukemia or after engraftment post-HCT and find relations between them and other parameters of iron metabolism, in the context of iron overload.

## 2. Materials and Methods

Study design. Pediatric patients with acute leukemia or after HCT, qualifying for the study, were analyzed for parameters of iron metabolism at a specific time point, in the context of blood transfusions and iron overload.

Patients. A total number of 85 patients (45 boys and 40 girls), with a median age of 7 (range 0–19) years, treated in two pediatric hematology and oncology centers between June 2019 and July 2020, were included into the study. Patients were enrolled into 4 groups: controls (group I), acute leukemia de novo (group II), acute leukemia after intensive treatment (group III), and patients after HCT (group IV) (Table 1). Serum samples were obtained at admission in group I, at the time of diagnosis in group II, within one month after consolidation therapy in group III, and one month after HCT in group IV. Children in groups III and IV were, after finalizing the respective periods of treatment, in an overall good condition, without signs of severe infection. Children with leukemia de novo were treated according to the AIEOP-BFM-ALL-2017 protocol or the AML-BFM-2019 protocol. Patients in group III completed intensive multiagent chemotherapy, usually complicated with frequent hematological adverse events and blood transfusions; during inclusion to the study, they were on oral maintenance chemotherapy. Patients who underwent HCT qualified according to the chemotherapy protocols. They were diagnosed with acute lymphoblastic leukemia (ALL, *n* = 6), acute myeloblastic leukemia (AML, *n* = 8), and other diagnoses including myelodysplastic syndrome (MDS, *n* = 1), severe aplastic anemia (SAA, *n* = 1), severe congenital neutropenia (SCN, *n =* 1), anaplastic large B-cell lymphoma (ALCL, *n =* 1), Ewing sarcoma (ES, *n =* 1), and neuroblastoma (NBL, *n* = 2), who underwent allogeneic transplantations in 18 (including 17 from matched unrelated donors, and 1 from family donor) cases and autologous ones in another 3. All transplantations were performed after myeloablative conditioning, except in 2 patients with SAA/SCN, who received reduced-intensity conditioning.

The control group was composed of healthy children with no history of any transfusions or hematological disorders. Overall, 60 (70.5%) children were transfused with concentrates of packed red blood cells (PRBC) (all patients in groups III and IV, none in group I, and 14/21 in group II). Patients received a median of 5 (range: 0–99) units of PRBC (including median of 1 unit in group II, median of 9 units in group III, and median of 23 units in group IV). At the time of analysis, 6/85 (7.1%) patients died. 

Collection of samples. Venous blood samples were collected from each participant under fasting conditions and placed into serum tubes (Becton Dickinson, Franklin Lakes, NJ, USA). Blood samples were allowed to clot for 30 min at room temperature and then were centrifuged for 20 min at 2000× *g* at room temperature. Serum was collected and stored at −80 °C until analyses. Serum samples from hemolyzed blood were excluded.

Iron metabolism parameters. To assess iron metabolism as comprehensively as possible, 14 laboratory parameters were analyzed in this study. They included three categories of markers: (1) parameters measuring functional and storage iron pools (NTBI, LPI, iron, transferrin, total iron-binding capacity (TIBC), ferritin, ferritin heavy chain (FTH1), and ferritin light chain (FTL)); (2) proteins regulating the absorption of iron and its release from the tissue stores (hepcidin (25-amino acid isoform), soluble hemojuvelin (sHJV), and soluble ferroportin-1 (sFNP-1)); (3) proteins regulating the erythropoietic activity of bone marrow (erythroferrone (ERFE), erythropoietin (EPO), and soluble transferrin receptor (sTfR)).

Determination of NTBI and LPI. Serum NTBI and LPI levels were determined at Savyon Diagnostics Ltd. (Ashdod, Israel) using fluorescence-based assays FeROS™eLPI and FeROS™LPI kit, respectively. The results are given in LPI units. NTBI and LPI were considered positive at ≥0.2 LPI units. The results below this value were regarded negative. 

Reagents. Determination of hepcidin (Intrinsic Hepcidin IDx™ ELISA Kit, ICE-007, Intrinsic Life Sciences, La Jolla, CA, USA), sHJV (ELISA Kit for Hemojuvelin (HJV), CEB995Hu, Cloud-Clone Corp., Katy, TX, USA), sFNP-1 (Human SLC40A1/Ferroportin-1 (Sandwich ELISA) ELISA Kit, LS-F33705, LifeSpan BioSciences, Inc., Seattle, WA, USA), ERFE (Intrinsic Erythroferrone IE™ ELISA Kit, ERF-001, Intrinsic Life Sciences, La Jolla, CA, USA), EPO (Erythropoietin Human ELISA, RAF013R, BioVendor-Laboratorni medicina a.s., Brno, Czech Republic), sTfR (sTfR Human ELISA (soluble Transferrin Receptor), RD194011100, BioVendor-Laboratorni medicina a.s., Brno, Czech Republic), FTH1 (Human FTH1/Ferritin Heavy Chain (Sandwich ELISA) ELISA Kit, LS-F26901, LifeSpan BioSciences, Inc., Seattle, WA, USA), FTL (Human FTL/Ferritin Light Chain (Sandwich ELISA) ELISA Kit, LS-F26902, LifeSpan BioSciences, Inc., Seattle, WA, USA), and transferrin (Transferrin Human ELISA Kit, EHTF, Thermo Fisher Scientific, Waltham, MA, USA) levels was conducted using a highly specific and sensitive enzyme-linked immunosorbent assay (ELISA) according to the manufacturer’s instructions. ELISA kits used in our study and their precise characterizations, including assay range, detection limit, and intra- and inter-assay precision, are shown in Table 2. Coefficients of variation (CVs) for all kits were lower than 10% for intra-assay and lower than 15% for inter-assay. Investigators who performed all the study’s assays were blind to clinical characteristics and patients’ outcomes.

Clinical parameters. Other iron status markers, including serum iron, ferritin, and TIBC, were measured using standard methods at the central hospital laboratory. Laboratory markers of inflammation, i.e., C-reactive protein (CRP) and procalcitonin (PCT), were also measured at the central hospital laboratory. 

Statistical analysis. The Wilcoxon U test and Mann–Whitney U test were used for non-categorical comparisons and the chi-square or Fisher exact test for categorical comparisons. The statistical level of significance was a 2-tailed *p*-value of < 0.05. The analysis was performed using the statistical package SPSS 25.0 (IBM, Armonk, NY, USA).

## 3. Results

### 3.1. NTBI/LPI and Other Parameters Measuring Functional and Storage Iron Pools

We first addressed whether NTBI and LPI are present in the blood of leukemic patients and the control group. NTBI was detected in all three patient groups, with the highest frequency in children after HCT (group IV), and this iron fraction was not found in the control group of healthy children (Table 3). The difference in incidence of NTBI between these groups (IV vs. controls) was statistically significant (*p* = 0.012). LPI was detected in the blood of all groups. Nevertheless, its presence was most common in children after HCT; this fraction was found in almost half of the group. The incidence of LPI in patients after HCT was significantly higher compared to controls (*p* = 0.018).

Then, we analyzed other parameters measuring functional and storage iron pools (iron, ferritin, transferrin, TIBC, FTH1, and FTL). The highest levels of iron and ferritin were found in patients after HCT, which corresponded to the highest values of the number of PRBC transfusions in this group of patients. Consequently, TIBC in patients after HCT or after chemotherapy for acute leukemia was lower than in healthy children (*p* < 0.001, for each group). The transferrin concentration was significantly decreased after HCT, when compared to patients after standard chemotherapy for acute leukemia (*p* = 0.011). On the other hand, FTH1 and FTL levels were not statistically different between the groups.

### 3.2. Proteins Regulating Absorption of Iron and Its Release from the Tissue Stores 

Differences in hepcidin and related protein levels (soluble hemojuvelin, sHJV; soluble ferroportin-1, FNP-1) were then assessed between the analyzed groups. Serum hepcidin was highly increased after HCT in comparison to the other groups. Additionally, the median concentration of FNP-1 was over 30% higher after HCT than in the other groups; however, the difference was not significant. The concentration of sHJV was highest in healthy children and gradually decreased after chemotherapy for acute leukemia (*p* = 0.025) and after HCT (*p* < 0.001 for control group; *p* = 0.007 for acute leukemia group). These results are summarized in Table 3.

### 3.3. Proteins Regulating Erythropoietic Activity of Bone Marrow

Subsequently, the relationship of bone marrow activity markers (EPO, ERFE, STfR) and phases of treatment of patients was investigated. The median EPO concentration was the highest in untreated patients with acute leukemia (group II) (*p* = 0.004 in comparison to group III, and *p* < 0.001 in comparison to other groups). On the other hand, no significant differences between groups were found for ERFE and STfR concentrations, although these values were also higher in the de novo acute leukemia group. The ERFE concentration in HCT patients was almost doubled in comparison to the group of patients after chemotherapy for acute leukemia. 

## 4. Discussion

In this study, we analyzed 14 biochemical parameters of iron metabolism in children after intensive chemotherapy of acute leukemia or after hematopoietic cell transplantation, when compared to healthy pediatric controls or children newly diagnosed with acute leukemia. We showed a variety of significant abnormalities in iron metabolism in these patients. The intensity of the pathology increased with the clinical pathology and intensity of treatment administered to these patients. Since treatment in these groups of patients was based on intensive multiagent chemotherapy, the most frequent adverse events included hematological consequences such as anemia, neutropenia, and thrombocytopenia. Frequent red cell transfusions are a standard approach in order to ensure patient safety.

The main findings of our study can be summarized in three aspects. Firstly, abnormalities in iron metabolism parameters are dependent on the intensity of treatment, being highest in children after HCT. Secondly, the intensity of chemotherapy results in an increase in NTBI and LPI, the most toxic forms of iron, occurring simultaneously with increased serum iron, ferritin, hepcidin, and ferroportin concentrations. Additionally, thirdly, based on our results, we can hypothesize the logical pathway of interactions between parameters in patients exposed to frequent blood transfusions due to leukemia or HCT. 

Results obtained in children might vary from those in adults, as the intensity of multiagent chemotherapy in pediatric protocols is usually much higher, and the profile of hematological toxicity is increased in this population [20]. This possibly results in more frequent transfusions of red cell concentrates, a higher risk of iron overload, and long-term sequelae, including lower overall survival [21,22,23,24]. Still, there are no comparative studies on iron metabolism abnormalities in children vs. adults undergoing chemotherapy/transplantation. One can expect that similar changes in iron metabolism parameters can occur in both age groups; however, it is anticipated that their intensity is deeper in children due to more intensive chemotherapy, complications, and blood transfusions.

This study was designed in order to cover gaps in the literature on pediatric patients. We determined the presence of NTBI and LPI simultaneously with other iron metabolism markers developing during chemotherapy in children undergoing anti-leukemic treatment or HCT. The measurement of NTBI and LPI in patients undergoing intensive multiagent chemotherapy for acute leukemia or HCT is of clinical relevance for both diagnosis and therapy as it could serve as a strong and reliable new indicator of iron overload. Possibly, it could also confirm the association between free iron and early toxicity and subsequent complications. Finally, these markers can be future potential targets for the use of iron chelators [10].

**Figure 1:**
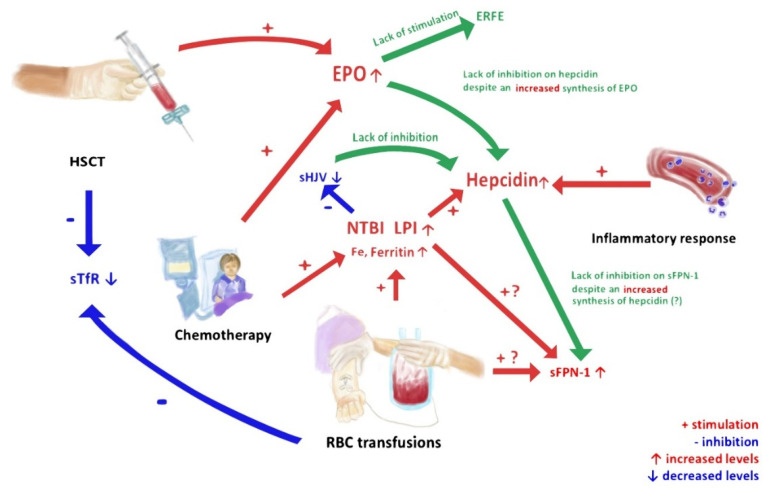
Flow chart of impact of anti-leukemic therapy and blood transfusions on mechanisms of iron overload metabolism.

All results obtained in our study can be grouped together in a pathophysiological network of changes in the expression of iron metabolism parameters with logical interactions resulting from clinical activity (Figure 1). The complexity of childhood leukemia therapy and hematopoietic cell transplantation affects the occurrence of disturbances in iron metabolism. Obviously, frequent blood transfusions lead to iron overload, and this is reflected in the high total levels of this element in the blood as well as the very high levels of ferritin. This results in the appearance of extremely toxic NTBI and LPI in the circulation, which may additionally be released from combinations with other blood compounds, as a result of chemotherapy administration. High iron levels and the presence of an increased inflammatory response induce hepatic hepcidin synthesis. We hypothesized that, at the same time, the high concentrations of iron led to a decrease in the release of HJV from cell membranes, which resulted in a reduction in the concentrations of sHJV, leading to a disturbance in the hepcidin–sHJV axis. Both HCT and chemotherapy affect the increased synthesis of EPO, which is a powerful regulator of the synthesis of ERFE and hepcidin. Despite the high levels of EPO, there is no stimulation of ERFE synthesis and no inhibition of hepcidin synthesis. EPO is unable to break the potency of two cardinal stimulators of hepcidin synthesis, namely, high blood iron levels and severe inflammation. An interesting observation was made regarding the relationship between hepcidin and its receptor FPN-1. The relationship between membrane FPN-1 and its serum form is not fully understood; however, the relationship is directly proportional. Although we did not find a statistically significant difference in the concentration of sFNP-1 between the subgroups, its highest concentration (with a median value almost 50% higher in comparison to the other groups) was recorded in group IV. This may be related to hyperferremia, which is known to enhance FNP expression. Thus far, only a few clinical studies have assessed the concentration of serum FNP-1. Our study is the first to use an ultra-sensitive enzyme immunoassay to evaluate the circulating pool of this protein. Still, the mechanism responsible for the lack of a negative feedback loop between hepcidin and sFPN-1 in hemato-oncological patients deserves further research.

The ferritin concentration progressively increased from groups I to IV. However, simultaneously, there were no significant differences in FTH and FTL chains. This discrepancy is a consequence of the structure and function of these proteins. Ferritin is composed of 24 heavy (H) and light (L) subunits, which, depending on the place of cellular synthesis, form a protein with different proportions between the subunits. FTL and FTH are subject to different translational and post-translational regulations. Various factors including cellular iron concentrations, the ongoing inflammatory process, and oxidative stress regulate the expression of FTL and FTH, often modifying it to a significantly different extent [25,26]. FTL and FTH fulfill different roles in iron metabolism. L subunits are responsible for iron storage and mineralization, while H subunits have a ferroxidase activity [27]. Hence, the precise relationships between these subunits, molecular regulations of their levels, their ability to form multimeric ferritin, and clinical significance are still matters of scientific debate.

Our study has some limitations. Although a large number of parameters were tested, and, in many cases, high statistical significance was found, the number of patients in each group is still not large enough to obtain a strong statistical power of this study. Additionally, this study did not analyze the differences in age groups: children vs. adults.

## 5. Conclusions

We showed an imbalance in iron metabolism, possibly increasing with the intensity of treatment with standard anti-leukemic chemotherapy and hematopoietic cell transplantation. In particular, the presence of NTBI and LPI increased with the intensity of anti-malignant treatment. This mostly affected children after transplantation. Iron metabolism abnormalities highly corresponded to the number of blood transfusions.

## Figures and Tables

**Table 1:** Patient characteristics.

Characteristics	Total (%) (*n* = 85)	Group I (*n =* 18)	Group II (*n =* 21)	Group III (*n =* 25)	Group IV (*n =* 21)
Age (years)					
Median (range)	7 (0–19)	8 (2–16)	7 (0–17)	5 (1–19)	8 (1–19)
Years: <10 vs. >10	59 (69.5):26 (30.5)	11 (61.1):7 (38.9)	16 (76.2):5 (23.8)	23 (92.0):2 (8.0)	9 (42.8):12 (57.2)
Gender					
male/female (%)	45 (52.9):40 (45.1)	8 (44.4):10 (55.6)	12 (57.2):9 (48.8)	13 (52.0):12 (48.0)	12 (57.2):9 (42.8)
Diagnosis					
ALL (%)	48 (56.4)	0	19 (90.4)	23 (92.0)	6 (28.6)
AML (%)	12 (0.1)	0	2 (9.5)	2 (8.0)	8 (38.1)
Other (%)	25 (0.3)	18 (100.0)	0	0	7 (33.3)
HCT (%)	21 (0.2)	0	0	0	21 (100)
PRBC transfusions					
>5 units (%)		0	2 (9.5)	23 (92.0)	21 (100)
>10 units (%)		0	1 (4.8)	11 (44.0)	19 (90.4)
>20 units (%)		0	0	2 (8.0)	15 (71.4)

ALL, acute lymphoblastic leukemia; AML, acute myeloblastic leukemia; HCT, hematopoietic cell transplantation; PRBC, packed red blood cell concentrate.

**Table 2:** The main features of enzyme-linked immunosorbent assay (ELISA) kits used in the current study.

Kit	Manufacturer	Assay Range	Detection Limit	Intra-Assay Coefficient of Variation (CV, %)	Inter-Assay Coefficient of Variation (CV, %)
Intrinsic Hepcidin IDx™ ELISA Kit (ICE-007)	Intrinsic Life Sciences (La Jolla, CA, USA)	5–250 ng/mL	2.5 ng/mL	2.4–4.0	2.6–3.8
Intrinsic Erythroferrone IE™ ELISA Kit (ERF-001)		0.16–10 ng/mL	0.02 ng/mL	4.7–6.7	7.0–14.9
ELISA Kit for Hemojuvelin (HJV) (CEB995Hu)	Cloud-Clone Corp. (Katy, TX, USA)	12.35–1000 ng/mL	4.93 ng/mL	<10	<12
Erythropoietin Human ELISA (RAF013R)	BioVendor-Laboratorni medicina a.s., (Brno, Czech Republic)	1.6–100 mIU/mL	0.14 mIU/mL	6.2	4.3
sTfR Human ELISA (soluble Transferrin Receptor) (RD194011100)		0.05–2 µg/mL	0.002 µg/mL	6.8	6.3
Transferrin Human ELISA Kit (EHTF)	Thermo Fisher Scientific (Waltham, MA, USA)	1.029–750 ng/mL	1 ng/mL	<10	<12
Human SLC40A1/Ferroportin-1 (Sandwich ELISA) ELISA Kit (LS-F33705)	LifeSpan BioSciences, Inc. (Seattle, WA, USA)	0.156–10 ng/mL	0.094 ng/mL	<8	<10
Human FTH1/Ferritin Heavy Chain (Sandwich ELISA) ELISA Kit (LS-F26901)		50–1000 pg/mL	1 pg/mL	<9	<10
Human FTL / Ferritin Light Chain (Sandwich ELISA) ELISA Kit (LS-F26902)		100–2500 pg/mL	1 pg/mL	<10	<12

**Table 3:** Differences between parameters of iron metabolism.

Parameters	Controls (Group I)	Acute Leukemia de Novo (Group II)	Acute Leukemia after Intensive Chemotherapy (Group III)	After HCT (Group IV)	*p*-Value
PRBC transfusions (units) median (range)	0 (0–0)	1 (0–10)	9 (2–35)	23 (6–99)	I vs. II; *p* < 0.001 I vs. III; *p* < 0.001 I vs. IV; *p* < 0.001 II vs. III; *p* < 0.001 II vs. IV; *p* < 0.001 III vs. IV; *p* < 0.001
NTBI positive (number)	0/18 (0%)	3/21 (14.3%)	3/25 (12.0%)	6/21 (28.6%)	I vs. II; *p* = 0.459 I vs. III; *p* = 0.058 I vs. IV; *p* = 0.012 II vs. III; *p* = 0.272 II vs. IV; *p* = 0.079 III vs. IV; *p* = 0.359
LPI positive (number)	2/18 (11.1%)	5/21 (23.8%)	6/25 (24.0%)	10/21 (47.6%)	I vs. II; *p* = 0.820 I vs. III; *p* = 0.398 I vs. IV; *p* = 0.018 II vs. III; *p* = 0.666 II vs. IV; *p* = 0.063 III vs. IV; *p* = 0.062
Serum iron (mg/dL) median (range)	69.2 (20.10–97.40)	125.05 (40.40–259.00)	103.6 (10.00–236.70)	128.40 (41.90–265.40)	I vs. II; *p* = 0.001 I vs. III; *p* = 0.007 I vs. IV; *p* = 0.001 II vs. III; *p* = 0.424 II vs. IV; *p* = 0.754 III vs. IV; *p* = 0.295
Transferrin (ng/mL) median (range)	32.11 (10.94–750.00)	32.95 (7.59–750.00)	38.00 (12.16–260.30)	23.36 (6.57–94.54)	I vs. II; *p* = 0.481 I vs. III; *p* = 0.109 I vs. IV; *p* = 0.371 II vs. III; *p* = 0.316 II vs. IV; *p* = 0.076 III vs. IV; *p* = 0.011
TIBC (µg/L) median (range)	356.00 (292.00–404.00)	276.50 (125.00–329.00)	262.50 (183.00–328.00)	234.00 (128.00–434.00)	I vs. II; *p* < 0.001 I vs. III; *p* < 0.001 I vs. IV; *p* < 0.001 II vs. III; *p* = 0.316 II vs. IV; *p* = 0.004 III vs. IV; *p* = 0.004
Ferritin (µg/L) median (range)	27.40 (11.00–73.30)	238.50 (14.20–1660.00)	739.00 (26.40–5278.00)	3670.00 (51.10–12,000.00)	I vs. II; *p* < 0.001 I vs. III; *p* < 0.001 I vs. IV; *p* < 0.001 II vs. III; *p* = 0.069 II vs. IV; *p* < 0.001 III vs. IV; *p* < 0.001
FTH1 (pg/mL) median (range)	16.45 (0.54–70.05)	24.45 (1.00–137.90)	18.81 (0.50–132.00)	22.76 (1.00–309.40)	I vs. II; *p* = 0.091 I vs. III; *p* = 0.481 I vs. IV; *p* = 0.528 II vs. III; *p* = 0.275 II vs. IV; *p* = 0.345 III vs. IV; *p* = 0.930
FTL (pg/mL) median (range)	94.65 (41.09–571.80)	129.30 (44.45–363.00)	113.30 (3.86–286.10)	117.00 (20.40–301.70)	I vs. II; *p* = 0.547 I vs. III; *p* = 0.990 I vs. IV; *p* = 0.918 II vs. III; *p* = 0.372 II vs. IV; *p* = 0.372 III vs. IV; *p* = 0.991
Hepcidin (ng/mL) median (range)	30.61 (14.55–468.20)	158.50 (21.69–738.60)	106.60 (17.26–383.20)	278.30 (22.15–1000.00)	I vs. II; *p* = 0.001 I vs. III; *p* = 0.013 I vs. IV; *p* < 0.001 II vs. III; *p* = 0.087 II vs. IV; *p* = 0.031 III vs. IV; *p* < 0.001
sHJV (ng/mL) median (range)	65.58 (49.02–91.47)	52.77 (27.33–88.23)	57.95 (24.78–136.80)	40.62 (18.34–98.41)	I vs. II; *p* = 0.029 I vs. III; *p* = 0.025 I vs. IV; *p* < 0.001 II vs. III; *p* = 0.635 II vs. IV; *p* = 0.051 III vs. IV; *p* = 0.007
FNP (pg/mL) median (range)	76.46 (41.94–251.90)	80.03 (29.12–924.70)	74.20 (38.57–618.20)	110.00 (37.69–1250.00)	I vs. II; *p* = 0.872 I vs. III; *p* = 0.990 I vs. IV; *p* = 0.212 II vs. III; *p* = 0.921 II vs. IV; *p* = 0.213 III vs. IV; *p* = 0.310
Erythroferrone (ERFE) (ng/mL) median (range)	0.83 (0.30–10.00)	1.29 (0.23–10.00)	0.69 (0.14–10.00)	1.26 (0.13–10.00)	I vs. II; *p* = 0.290 I vs. III; *p* = 0.191 I vs. IV; *p* = 0.837 II vs. III; *p* = 0.024 II vs. IV; *p* = 0.279 III vs. IV; *p* = 0.494
EPO (mIU/mL) median (range)	5.26 (0.45–12.73)	44.85 (1.81–100.00)	12.73 (5.31–100.00)	12.32 (3.10–100.00)	I vs. II; *p* < 0.001 I vs. III; *p* < 0.001 I vs. IV; *p* < 0.001 II vs. III; *p* = 0.004 II vs. IV; *p* < 0.001 III vs. IV; *p* = 0.372
STfR (µg/mL) median (range)	0.12 (0.07–0.29)	0.11 (0.09–0.52)	0.11 (0.08–0.71)	0.09 (0.07–0.32)	I vs. II; *p* = 0.340 I vs. III; *p* = 0.918 I vs. IV; *p* = 0.020 II vs. III; *p* = 0.480 II vs. IV; *p* = 0.024 III vs. IV; *p* = 0.012
CRP (mg/L) median (range)	0.49 (0.16–2.34)	6.68 (0.37–156.50)	0.50 (0.16–36.37)	3.51 (0.22–297.54)	I vs. II; *p* < 0.001 I vs. III; *p* = 0.739 I vs. IV; *p* < 0.001 II vs. III; *p* < 0.001 II vs. IV; *p* = 0.580 III vs. IV; *p* < 0.001
PCT (ng/mL) median (range)	0.02 (0.02–0.10)	0.10 (0.01–2.82)	0.05 (0.00–0.36)	0.16 (0.02–11.47)	I vs. II; *p* < 0.001 I vs. III; *p* = 0.002 I vs. IV; *p* < 0.001 II vs. III; *p* = 0.005 II vs. IV; *p* = 0.669 III vs. IV; *p* = 0.002

PRBC, packed red blood cell concentrate; NTBI, non-transferrin-bound iron; LPI, labile plasma iron; sHJV, soluble hemojuvelin; sFNP-1, soluble ferroportin-1; ERFE, erythroferrone; EPO, erythropoietin; sTfR, soluble transferrin receptor; TIBC, total iron-binding capacity; FTH, ferritin heavy chain; FTL, ferritin light chain; CRP, C-reactive protein; PCT, procalcitonin; HCT, hematopoietic cell transplantation.

## Data Availability

The data presented in this study are available on request from the corresponding author. The data are not publicly available due to privacy restrictions.
